# The role of TGF-β1 in chronic multilobar segmental bronchial stenosis and advances in targeted drug research

**DOI:** 10.3389/fphar.2025.1649570

**Published:** 2025-10-27

**Authors:** Mingjun Wu, Qian Yang, Youcheng Xie, Yan Hou, Qingliang Xue

**Affiliations:** 1 Department of Respiratory and Critical Care Medicine, 940th Hospital of Joint Logistic Support Force of Chinese People’s Liberation Army, Lanzhou, Gansu, China; 2 Department of Plateau Medicine, 940th Hospital of Joint Logistic Support Force of Chinese People’s Liberation Army, Lanzhou, Gansu, China; 3 Central Sterile Supply Department, 940th Hospital of Joint Logistic Support Force of Chinese People’s Liberation Army, Lanzhou, Gansu, China

**Keywords:** chronic multilobar segmental bronchial stenosis, transforming growth factor-β1, Smad signaling pathway, targeted therapy, airway remodeling

## Abstract

Chronic multilobar segmental bronchial stenosis (CMBS) is a chronic inflammatory airway disease characterized by stenosis across multiple lobar and segmental bronchi, primarily diagnosed via bronchoscopy. Epidemiologically, its prevalence exhibits significant regional variation, ranging from 0.1% to 22.5%, with higher rates observed in developing countries, rural populations, women, and individuals with a history of tuberculosis. Clinically, CMBS manifests as progressive dyspnea, chronic cough, recurrent pulmonary infections, and obstructive ventilatory dysfunction that is typically poorly responsive to bronchodilators. Radiologically, high-resolution computed tomography (HRCT) reveals characteristic bronchial wall thickening, luminal narrowing, and often associated mediastinal or peribronchial calcified lymph nodes. Long-term exposure to biomass fuel smoke (e.g., from wood or coal combustion), is established as a major etiological factor. Other significant risk factors include prior tuberculosis infection, and occupational exposures to inhalable irritants like silica dust in mining or textile workers. Despite its considerable global disease burden, the precise pathogenesis remains elusive. Research has identified transforming growth factor-β1 (TGF-β1) as a pivotal regulator of airway remodeling in various chronic respiratory diseases, such as asthma and chronic obstructive pulmonary disease (COPD). Notably, animal models of chronic biomass smoke exposure demonstrate a significant correlation between upregulated TGF-β1 expression and a distinct airway remodeling phenotype, suggesting its potential involvement in the pathological progression of CMBS. Accumulating evidence indicates that TGF-β1 mediates airway remodeling through multiple intricate mechanisms, including immune dysregulation, fibroblast activation and proliferation, aberrant extracellular matrix (ECM) deposition, epithelial-mesenchymal transition (EMT), and pathological vascular remodeling. In recent years, groundbreaking progress has been made in research on therapeutics targeting the TGF-β1 signaling pathway, including monoclonal antibodies (e.g., Fresolimumab), small molecule kinase inhibitors (e.g., Galunisertib, TEW-7197), and novel targeted delivery systems. This review systematically summarizes the molecular mechanisms of TGF-β1 in CMBS airway remodeling and the advances in the development of targeted drugs. Furthermore, it proposes future research directions focused on CMBS-specific applications, such as validating these therapeutics in preclinical CMBS models, developing inhaled formulations for localized delivery, establishing biomarker-driven patient stratification, and exploring combination therapies with anti-fibrotic agents. This aims to provide a comprehensive theoretical foundation for elucidating the disease’s pathology and developing novel, precise diagnostic and therapeutic strategies for CMBS.

## 1 Introduction

Chronic multilobar segmental bronchial stenosis (CMBS) is a chronic inflammatory airway disease characterized by multiple fibrotic stenoses in the lobar and segmental bronchi. Its clinical diagnosis relies on bronchoscopy, typically revealing bronchial stenosis or obliteration accompanied by mucosal pigmentation (Chung et al., 1998; Kim et al., 2014). Epidemiological studies indicate that the incidence of CMBS ranges from 0.1% to 22.5%, with significant geographical and demographic variations; it is more prevalent in developing countries, rural areas, women, and populations with a high burden of tuberculosis (TB) (Mirsadraee, 2014). Long-term exposure to biomass fuel smoke (e.g., from wood or coal) is a primary causative factor, and the risk increases with prolonged exposure duration (Kim et al., 2000; Jamaati et al., 2017). Tuberculosis infection is another significant risk factor, with approximately 27%–60% of patients having co-existing TB infection, often associated with reactivation of previously treated lesions (Jamaati et al., 2017; Das et al., 2021; Kim et al., 2009)^.^ Occupational exposure further exacerbates the risk, as textile and mining workers exhibit a higher prevalence of CMBS than the general population, primarily due to chronic airway inflammatory damage induced by inhalable dusts (e.g., silicates) (Dutta et al., 2024; Wynn et al., 2008).

CMBS commonly manifests as progressive dyspnea, recurrent infections, and obstructive ventilatory dysfunction, with poor response to bronchodilators. Pulmonary function tests often indicate a decline in parameters such as FEV1 and FEV1/FVC. Imaging characteristics include bronchial stenosis with wall thickening, and calcified lymph nodes in the mediastinum or peribronchial regions. However, due to its high clinical similarity to chronic obstructive pulmonary disease (COPD) and a lack of physician awareness, CMBS is frequently misdiagnosed as COPD. A study from South Korea revealed that among patients hospitalized for acute exacerbation of COPD, the prevalence of CMBS was as high as 25% (Kim et al., 2014), underscoring its significant risk for being overlooked or misdiagnosed.

Globally, biomass fuel remains the primary domestic energy source for approximately 3 billion people, particularly in Asia and Eastern Europe. Increased poverty resulting from the COVID-19 pandemic may further intensify reliance on biomass fuels (Li S. et al., 2024; Stolz et al., 2022), suggesting a potential continued rise in CMBS prevalence and establishing it as a pressing public health concern. Currently, there is no curative treatment for CMBS; existing therapies only alleviate symptoms without halting or reversing disease progression. Therefore, in-depth investigation into its pathogenesis holds significant clinical importance.

In recent years, transforming growth factor-β1 (TGF-β1), a pleiotropic cytokine, has been recognized to play a critical role in the airway remodeling process of chronic respiratory diseases such as asthma, COPD, and pulmonary fibrosis (Hamidi et al., 2017; Li Y. et al., 2024). Autopsy pathology of CMBS has revealed airway remodeling features similar to those observed in these diseases (Ramírez-Venegas et al., 2019). Animal experiments have further confirmed that chronic biomass smoke exposure can induce upregulated TGF-β1 expression in rat lung tissue alongside a typical airway remodeling phenotype (Wang et al., 2022), providing direct evidence for the pivotal role of TGF-β1 in CMBS pathogenesis. TGF-β1 is likely involved in the pathogenesis of CMBS through multiple mechanisms, including modulating immune responses, promoting fibroblast activation and extracellular matrix (ECM) metabolic dysregulation, inducing epithelial-mesenchymal transition (EMT), and facilitating pathological vascular remodeling. Further elucidation of the molecular regulatory mechanisms of TGF-β1 in CMBS will not only enhance the understanding of its pathophysiological basis but also provide crucial directions for developing targeted therapeutic strategies.

## 2 Biological functions of TGF-β1 and its signal transduction mechanisms

TGF-β1 is a key member of the TGF-β superfamily, which comprises 33 structurally homologous and functionally related polypeptide growth factors. TGF-β1 plays a central role in regulating critical biological processes such as cell differentiation, proliferation cycle, extracellular matrix remodeling, and tissue repair (Wrighton et al., 2009). Notably, among the three homologous TGF-β isoforms (TGF-β1, TGF-β2, and TGF-β3), TGF-β1 possesses the broadest tissue expression profile and exhibits the most extensive biological effects (Ojiaku et al., 2017). TGF-β1 activates downstream signaling networks by forming a heteromeric receptor complex composed of type I (TβRI) and type II (TβRII) transmembrane serine/threonine kinase receptors. Both receptor types belong to the dual-specificity kinase family, and their ligand-binding properties determine the specificity of the signaling pathway and the selection of downstream effector molecules (Woo et al., 2021). Current research confirms that TGF-β1 signal transduction primarily relies on the Smad protein-mediated canonical pathway and alternative non-Smad dependent pathways.

### 2.1 Smad-dependent signaling pathway

**FIGURE 1:**
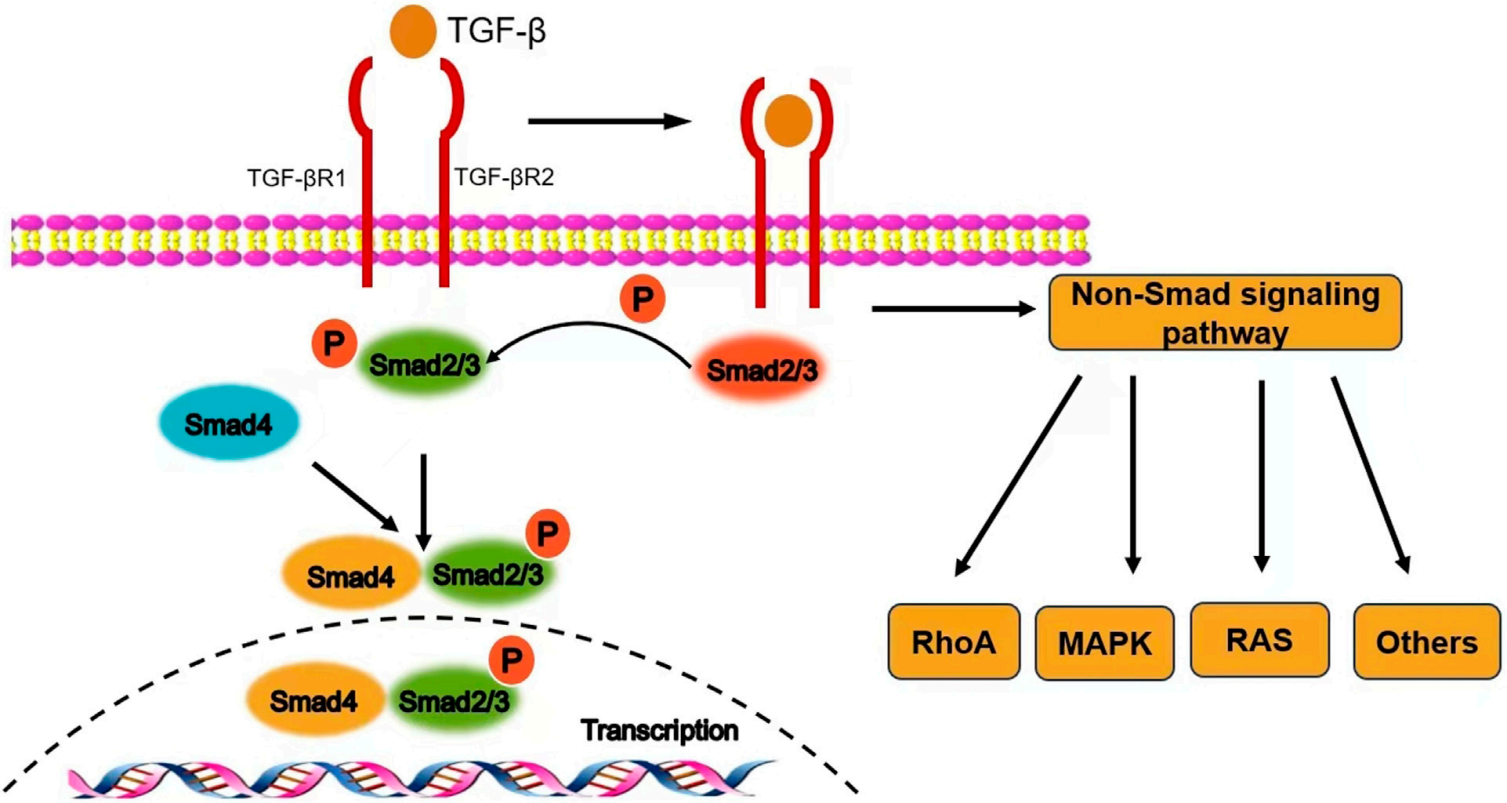
The signal transduction mechanism of TGF-β1.

As the core mechanism of TGF-β1 signal transduction, the Smad pathway comprises three functionally distinct protein families: receptor-activated Smads (R-Smads, including Smad2/3), common-mediator Smads (co-Smads, Smad4), and inhibitory Smads (I-Smads, Smad6/7). Following ligand-receptor binding, TβRII phosphorylates and activates TβRI, which subsequently activates R-Smads through phosphorylation of their C-terminal SSXS motif. The phosphorylated R-Smads form a heterotrimeric complex with co-Smad, which translocates into the nucleus. There, it interacts with chromatin remodeling and transcriptional regulatory elements to activate or repress target gene expression (Gao et al., 2024) (Figure 1). Notably, Davis et al. discovered that R-Smads can form functional complexes with microRNAs, participating in the post-transcriptional regulation of gene expression by inducing RNA degradation, revealing a multi-layered regulatory mechanism within the Smad signaling network (Davis-Dusenbery and Hata, 2011).

### 2.2 Negative regulatory mechanisms of the signaling pathway

I-Smads inhibit the phosphorylation of R-Smads by competitively binding to TβRI, while concurrently cooperating with Smad ubiquitination regulatory factors to promote the ubiquitination and degradation of the receptor complex, thereby achieving dynamic negative regulation of TGF-β1 signaling. This bidirectional regulatory mechanism ensures the precision and reversibility of signal transduction.

### 2.3 Non-Smad dependent signaling pathways

In addition to the canonical Smad pathway, TGF-β1 can activate various alternative signaling cascades, including the mitogen-activated protein kinase (MAPK) cascade, the phosphoinositide 3-kinase (PI3K)/protein kinase B (Akt) pathway, and cytoskeletal remodeling mediated by Rho family small GTPases (e.g., Rac/Cdc42) (Hamidi et al., 2017).

## 3 Mechanism of action of TGF-β1 in CMBS airway remodeling

**FIGURE 2:**
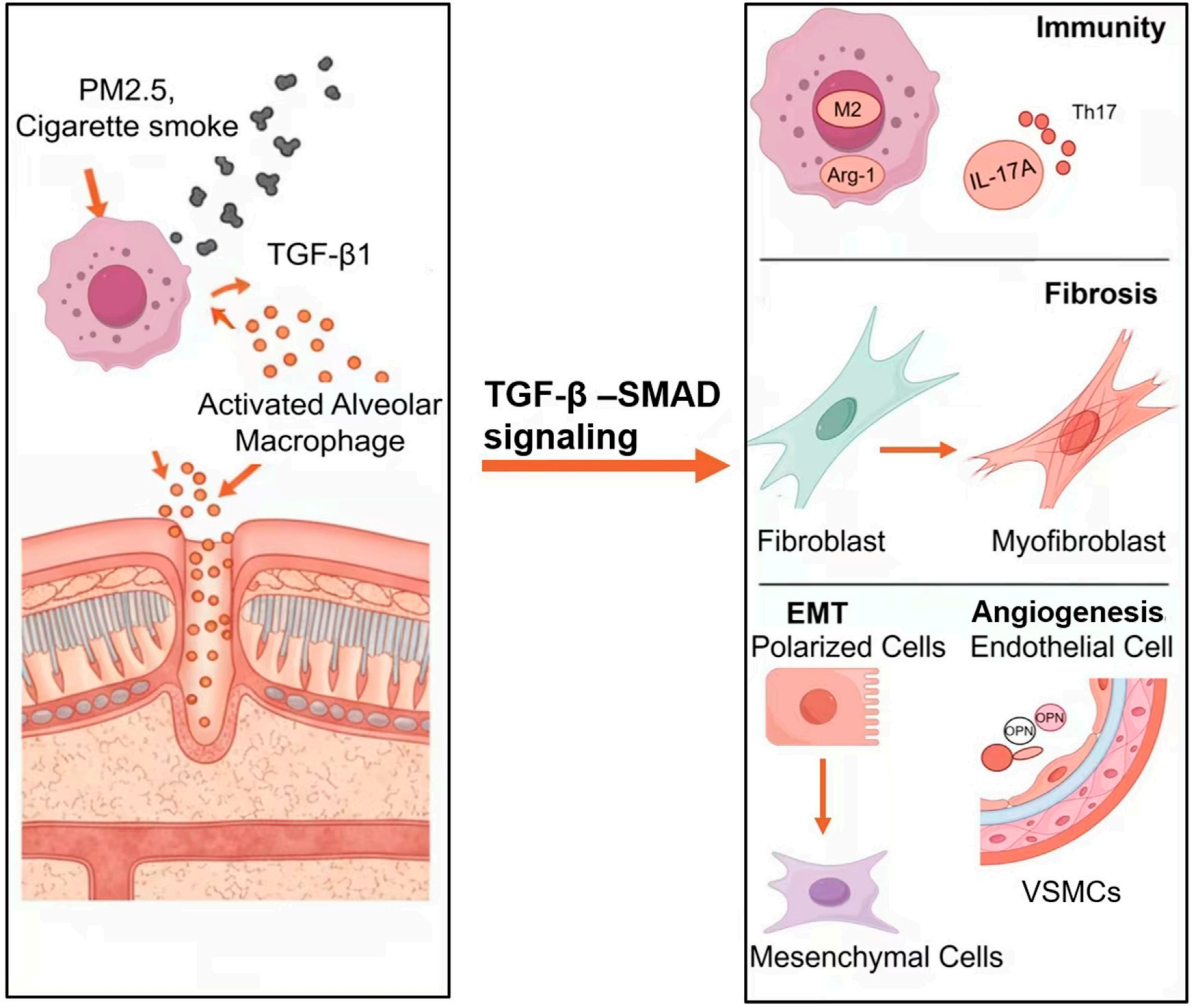
The mechanism of TGF-β1 in CMBS airway remodeling.

Autopsy pathological analyses (Rivera et al., 2008) reveal that characteristic pathological alterations in CMBS predominantly involve significant bronchial wall thickening, manifesting as basement membrane thickening, peribronchiolar fibrosis, aberrant ECM deposition, and bronchiectasis, while emphysematous changes are relatively uncommon. Multiple studies indicate that chronic biomass smoke exposure activates the TGF-β1/Smad3 signaling axis, leading to increased airway wall thickness and destruction of alveolar structure (Hamidi et al., 2017; Wang et al., 2022). Notably, rat models of biomass smoke exposure established by Li S. et al. (2024) and Wang et al. (2022) demonstrated that the airway remodeling phenotype after 6 months of exposure was significantly correlated with upregulated expression of TGF-β1 and its phosphorylated Smad3 (p-Smad3), suggesting this signaling pathway may play a central regulatory role in the pathological progression of CMBS. Current research has unveiled that TGF-β1 drives airway remodeling through the following mechanisms (Figure 2).

### 3.1 Immune dysregulation

The chronic immune-inflammatory response triggered by biomass smoke exposure is a significant driver of airway remodeling. Alveolar macrophages (AMs), as key regulators of pulmonary immune homeostasis, undergo phenotypic polarization (switching from pro-inflammatory M1 to anti-inflammatory M2), which is closely associated with the dynamic expression of TGF-β1 (Gordon, 2003). Experiments by Wang et al. (2022) showed that AMs release pro-inflammatory factors causing tissue damage during the initial phase of biomass smoke exposure, whereas sustained exposure for 6 months was accompanied by a significant increase in TGF-β1 and enhanced M2 polarization. M2 macrophages highly express arginase-1 (Arg-1) and chitinase-3-like protein 3 (Ym-1). They not only suppress inflammatory responses but also directly participate in fibroblast activation and ECM deposition by secreting pro-fibrotic factors such as TGF-β1 and PDGF (Mills et al., 2000), indicating that TGF-β1 contributes to the remodeling process by regulating macrophage phenotypic switching.

Furthermore, the role of the Th17/IL-17A axis in airway remodeling has garnered significant attention. Biomass smoke can promote dendritic cell maturation via the TGF-β1/STAT3 pathway, thereby inducing the differentiation of naïve CD4^+^ T cells into Th17 cells, which secrete IL-17A (Solleiro-Villavicencio et al., 2015; Pu et al., 2021). IL-17A, acting as a core mediator of both pro-inflammatory and pro-fibrotic effects, can directly activate fibroblasts, upregulate the expression of collagen genes such as COL1A1 and COL3A1, and enhance metalloproteinase-9 (MMP-9) activity to promote ECM remodeling. Notably, IL-17A can also amplify TGF-β1 signaling through a positive feedback loop: on one hand, IL-17A induces the expression of TGF-β1 precursor by activating the NF-κB pathway; on the other hand, it reinforces TGF-β1/Smad signal transduction by suppressing Smad7 expression, thereby establishing a pro-fibrotic cascade (Huo et al., 2021).

Recent studies have also elucidated the synergistic roles of innate lymphoid cells (ILCs) and regulatory T cells (Tregs) in immune dysregulation. ILC2s increase following biomass smoke exposure and collaborate with TGF-β1 by secreting IL-13 to promote fibroblast transformation (Rosário Filho et al., 2021). Although Tregs possess anti-inflammatory functions, in a TGF-β1-dominated microenvironment, they may indirectly contribute to the fibrotic process by expressing glycoprotein A repetitions predominant (GARP), which facilitates the activation of latent TGF-β1 (Moreau et al., 2022). The dysregulation of this immune cell network collectively establishes a chronic inflammatory microenvironment centered around TGF-β1 with multicellular interactions, ultimately leading to irreversible airway structural remodeling.

### 3.2 Fibroblast activation and ECM metabolic dysregulation

Aberrant fibroblast activation and excessive ECM deposition are core pathological features of airway remodeling. Studies indicate that TGF-β1 plays a key role in airway structural reconstruction by inducing fibroblast proliferation, myofibroblast transdifferentiation, and the overproduction of matrix components such as collagen (Paw et al., 2023; Guan et al., 2020). Consequently, TGF-β1-induced fibroblast activation models have become important *in vitro* tools for studying airway remodeling. Under physiological conditions, lung fibroblasts maintain low proliferative activity, but can transition to a high-secretory phenotype upon stimulation by an inflammatory microenvironment. Experimental evidence shows that human lung fibroblasts exposed to biomass smoke extract exhibit upregulated expression of TGF-β1 and type I/III collagen within just 3 h, suggesting that environmental exposure can drive fibroblast activation via the TGF-β1 pathway (Recillas-Román et al., 2021). Notably, the dynamic imbalance between ECM synthesis and degradation is a crucial mechanism leading to excessive matrix accumulation. TGF-β1 contributes to this process through dual regulation: it promotes the biosynthesis of ECM components like collagen, while simultaneously inhibiting the degradative activity of matrix metalloproteinases (MMPs), thereby disrupting ECM metabolic equilibrium (Savin et al., 2023; Hough et al., 2020; Murray and Wynn, 2011).

The canonical Smad signaling pathway is the primary molecular mechanism through which TGF-β1 mediates airway remodeling. Research by Song et al. found that TGF-β1 can facilitate the phosphorylation and nuclear translocation of STAT3/6 by forming a Smad3/JAKs/STAT3/6/EPRS multi-protein complex, thereby activating the transcriptional program of ECM-related genes (Song et al., 2018). Furthermore, microRNA-21 was shown in an asthma model to regulate collagen and pro-fibrotic gene expression via the TGF-β1/Smad7 signaling axis, and its inhibitor significantly alleviated the degree of airway remodeling (Hur et al., 2021). Recent studies have revealed the involvement of various non-canonical pathways in TGF-β1-mediated fibrosis, including the MAPK cascade, the PI3K/Akt pathway, and the Rac/Cdc42 pathway, among others (Hamidi et al., 2017).

### 3.3 Epithelial-mesenchymal transition (EMT)

EMT is a core pathological process in airway remodeling, characterized by the loss of epithelial cell polarity and the acquisition of a mesenchymal phenotype. Research demonstrates that TGF-β1 has a potent EMT-inducing effect on alveolar and bronchial epithelial cells, a process that can be recapitulated in *in vitro* culture models and animal experiments (Willis et al., 2005; Zhang et al., 2009). He et al. (2017) established a rat model exposed to biomass smoke for 7 months and found a significant positive correlation between serum TGF-β1 levels and the expression of bronchial epithelial mesenchymal markers (FSP1, vimentin), providing direct evidence that biomass smoke promotes EMT via the TGF-β1 pathway, leading to airway remodeling. Current research has elucidated multiple molecular mechanisms through which TGF-β1 induces EMT.

c-Jun N-terminal kinase 1 (JNK1) is a critical regulatory node in TGF-β1-mediated EMT. Experiments confirmed that JNK1 cooperatively activates EMT-related transcriptional programs by forming a phosphorylation complex with Smad3. In JNK1 knockout mice, the pulmonary fibrosis and airway remodeling phenotypes induced by bleomycin or house dust mite were completely abolished, indicating the necessity of this kinase in the EMT process (van der Velden et al., 2020). TGF-β1 forms a positive feedback loop with matrix MMP-9. TGF-β1 induces MMP-9 expression, which promotes basement membrane degradation, while MMP-9 can further activate the signaling pathway by releasing latent TGF-β1. In the airways of COPD patients, this loop leads to abnormal deposition of EMT-derived mesenchymal cells, exacerbating airway wall fibrosis (Cabrera-Benítez et al., 2012). Mitogen-activated protein kinase 19 (MAPK19), a newer member of the MAPK family, was found to synergize with *Mycobacterium bovis* BCG to activate the TGF-β/Smad2 pathway, inducing EMT in type II alveolar epithelial cells. Blocking this pathway with the specific inhibitor LY2109761 significantly reduced the proportion of EMT cells and collagen deposition (Xia et al., 2024).

Although the aforementioned mechanisms have been validated in various pulmonary diseases, the specific regulatory network of TGF-β1-mediated EMT in CMBS remains to be fully elucidated. Future research should integrate single-cell sequencing and spatial transcriptomic technologies to decipher the spatiotemporal dynamics of airway epithelial EMT and identify its pivotal molecular drivers under biomass smoke exposure.

### 3.4 Pathological vascular remodeling

Bronchoscopy in CMBS patients often reveals features such as tortuous and hyperplastic mucosal vasculature, increased wall fragility, and a tendency for bleeding, indicating that vascular remodeling is a significant pathological alteration (Lu et al., 2022). Animal experiments show that biomass smoke exposure for 6 months concurrently upregulates the expression of both TGF-β1 and vascular endothelial growth factor (VEGF) in rat lung tissue (Wang et al., 2022), suggesting that TGF-β1 may play a central regulatory role in CMBS-associated vascular remodeling. At the molecular level, TGF-β1 induces the transition of vascular smooth muscle cells (VSMCs) from a contractile to a synthetic phenotype via Smad pathways (e.g., activation of the Smad2/3-Smad4 complex) and non-Smad pathways (e.g., JNK and ERK). This is characterized by downregulation of α-smooth muscle actin (α-SMA) and upregulation of pro-proliferative proteins such as osteopontin (OPN), vimentin, and proliferating cell nuclear antigen (PCNA) (Wang et al., 2022; Da et al., 2023).

Significant crosstalk exists between the TGF-β1 and VEGF signaling pathways: on one hand, TGF-β1 upregulates VEGF-A expression through Smad3-mediated transcriptional activation; on the other hand, it enhances the expression and phosphorylation of VEGFR2, thereby amplifying pro-angiogenic signals (Yan et al., 2020). Notably, TGF-β1 differentially regulates members of the VEGF family; for instance, it inhibits VEGF-D expression in human lung fibroblasts via the JNK pathway, potentially affecting the balance between angiogenesis and lymphangiogenesis (Cui et al., 2014).

Although research directly linking biomass smoke to pulmonary vascular remodeling is limited, cigarette smoke models provide strong corroborative evidence. Cigarette smoke extract can induce abnormal proliferation of pulmonary artery smooth muscle cells (PASMCs) via the ERK pathway and promote VSMC migration through upregulation of OPN in animal models (Su et al., 2021; Zhou et al., 2024). Both biomass smoke and cigarette smoke are rich in PM2.5 and may cause vascular pathology through shared mechanisms such as oxidative stress and chronic inflammation. Recent studies suggest that biomass combustion smoke may induce hypermethylation of the EDN1 gene, regulating endothelin-1 (ET-1) levels and participating in vascular remodeling (García-Carmona et al., 2025). Macrophages also play a key role in regulating the vascular microenvironment, either by secreting TGF-β1 or through pathways such as FOXO1-SEMA3C (Niimi et al., 2024).

In summary, TGF-β1 can drive vascular remodeling in CMBS by regulating VSMC phenotypic switching and VEGF signaling through a multi-pathway network. Biomass smoke may share some pathogenic mechanisms with cigarette smoke, but its specific components and epigenetic regulatory pathways require further investigation.

## 4 Current status of TGF-β1-targeting drugs and their therapeutic potential in CMBS

Aberrant activation of the TGF-β1 signaling pathway induced by biomass smoke exposure is a key mechanism in CMBS airway remodeling. Therefore, targeting this pathway represents a promising therapeutic strategy. Although no studies have directly investigated the application of TGF-β1-targeting drugs in CMBS, the bronchial wall fibrosis observed in CMBS shares high similarity in its core pathological mechanisms with other fibrotic diseases driven by TGF-β1, such as pulmonary fibrosis and systemic sclerosis. The positive outcomes achieved with TGF-β1 inhibitors in other fibrotic models provide a strong theoretical basis for extrapolating their therapeutic potential in CMBS and warrant future preclinical studies for further validation. The following section will focus on reviewing the research progress of TGF-β1-targeting drugs, including monoclonal antibodies and small molecule kinase inhibitors, in fibrotic diseases, and further discuss their potential application value in CMBS treatment (Table 1).

**TABLE 1:** TGF-β pathway-targeting drugs and their potential in CMBS therapy.

Drug name	Clinical stage (indication)	Efficacy	Safety	Potential for CMBS application
Fresolimumab (Monoclonal Antibody)	Clinical Research (Systemic Sclerosis, Primary FSGS)	Reduces TGF-β gene expression; decreases myofibroblasts; slows renal function decline	Reversible skin toxicity	Broad-spectrum neutralization can comprehensively block biomass smoke-induced fibrosis; reversible dermal toxicity supports potential for long-term use
Galunisertib (Small Molecule Kinase Inhibitor)	Preclinical (Liver, Kidney, Skin Fibrosis)	Reduces collagen deposition; inhibits fibrosis progression	No cardiotoxicity	Suitable for inhaled formulation development, acting directly on the airways for local treatment to inhibit bronchial wall fibrosis, with low systemic exposure risk
TEW-7197 (Small Molecule Kinase Inhibitor)	Preclinical (Liver, Kidney, Lung Fibrosis, Asthma)	Inhibits fibrosis; inhibits angiogenesis (asthma models)	Well-tolerated	Targeted delivery systems (e.g., engineered stem cells) provide a novel approach for precise drug delivery to CMBS airway lesion sites
LY3200882 (Small Molecule Kinase Inhibitor)	Phase I (Solid Tumors)	Enhances efficacy of radiotherapy and immunotherapy	Grade 3 AEs in combination therapy	Its smart-responsive delivery technology concept can be adapted to develop intelligent drug release systems targeting CMBS airway inflammation for precise therapy

### 4.1 Monoclonal antibodies

Fresolimumab is a broad-spectrum, fully human monoclonal antibody that neutralizes TGF-β1, β2, and β3. In fibrotic disease research, this drug significantly reduced the expression of TGF-β-regulated genes (SERPINE1, CTGF) and decreased dermal myofibroblast infiltration in patients with systemic sclerosis (Rice et al., 2015). It also demonstrated good safety and potential for slowing renal function decline in patients with primary focal segmental glomerulosclerosis (Trachtman et al., 2011; Vincenti et al., 2017). Its safety profile, characterized by reversible skin toxicity, supports the possibility of long-term administration. Given that CMBS is also characterized by TGF-β1-driven fibrosis, fresolimumab holds promise for blocking biomass smoke-induced airway fibrosis by neutralizing multiple TGF-β isoforms. Its safety profile with reversible skin toxicity also supports the potential for long-term use.

### 4.2 Small molecule kinase inhibitors

Galunisertib (LY2157299) is an oral TβRI kinase inhibitor that blocks TGF-β signal transduction by competitively binding to the ATP-binding site of TβRI, thereby inhibiting Smad2/3 phosphorylation. Galunisertib shows great promise in treating fibrotic diseases. Studies have confirmed its significant inhibitory effects on pathological processes such as dermal fibroblast fibrosis, liver fibrosis, and renal fibrosis (Peterson et al., 2022; Hammad et al., 2018; van Leeuwen et al., 2024). Its favorable safety profile, particularly the absence of significant cardiovascular toxicity, offers an advantage for long-term use in chronic respiratory diseases. Based on these findings, galunisertib holds potential for directly targeting the airways via local inhalation administration to inhibit the fibrotic process in the bronchial walls of CMBS patients.

TEW-7197 (vactosertib) is another highly selective TβRI inhibitor. Preclinical studies have confirmed its efficacy in inhibiting fibrosis progression in models of liver, kidney, and pulmonary fibrosis (Park et al., 2015). Recent research has explored its potential for respiratory application. Surface-engineered mesenchymal stem cells constructed via bioconjugation technology (combining TEW-7197 and linifanib) effectively suppressed subepithelial fibrosis and angiogenesis in an asthma model (He et al., 2024). This delivery strategy offers a novel approach for CMBS treatment, whereby TEW-7197 could be delivered directly to the airway lesions via a targeted drug delivery system, maximizing therapeutic efficacy while minimizing systemic exposure.

LY3200882 is a dual TβRI/ALK5 inhibitor. Preclinical studies have confirmed its ability to enhance the anti-tumor immune response to radiotherapy (Hamon et al., 2022). Although there are no published reports on this compound in fibrotic diseases to date, its mechanism of action still provides important rationale for targeting the TGF-β signaling pathway. Notably, a tumor microenvironment-responsive drug delivery system developed by Li et al. (2022) offers an innovative concept for local treatment strategies in CMBS. Drawing on such advanced technologies, future efforts could design intelligent responsive drug delivery systems tailored to the characteristics of the airway inflammatory microenvironment in CMBS (e.g., specific protease activity or pH changes). This would enable the precise and controlled release of TGF-β inhibitors at the site of bronchial stenosis, thereby improving the efficiency and safety of targeted therapy.

### 4.3 Prospects and challenges

Although TGF-β-targeting drugs show promising therapeutic potential in fibrotic diseases, their application in CMBS faces several challenges. Firstly, current research on TGF-β pathway inhibition primarily focuses on diseases like cancer and fibrosis; their efficacy, optimal dosing, and long-term safety in CMBS remain unclear and urgently require evaluation through rigorous preclinical and clinical studies. Secondly, the route of administration needs optimization. Developing inhaled formulations could achieve local drug enrichment in the airways, enhance targeting, and reduce systemic exposure risk, thereby improving the risk-benefit ratio of treatment. Furthermore, it is necessary to establish patient stratification strategies based on imaging characteristics (e.g., degree and distribution of bronchial stenosis) and molecular biomarkers (e.g., levels of TGF-β1, p-Smad3) to accurately identify CMBS subpopulations most likely to benefit from anti-TGF-β therapy. Concurrently, combination regimens of TGF-β inhibitors with existing anti-fibrotic drugs (e.g., pirfenidone) should be actively explored to achieve synergistic inhibition of the fibrotic process. Future research should prioritize the establishment of CMBS animal models that simulate biomass smoke exposure. Systematically validating the efficacy and safety of TGF-β-targeting drugs in such models will provide a solid theoretical foundation for subsequent clinical trials.

## 5 Conclusion

CMBS is a chronic inflammatory airway disease that is frequently misdiagnosed and lacks effective treatments, characterized primarily by multiple fibrotic stenoses in the lobar and segmental bronchi. This review systematically elaborates on the significant role of TGF-β1 as a core driver in CMBS airway remodeling through multiple mechanisms, including modulating immune responses, promoting fibroblast activation and ECM metabolic dysregulation, inducing epithelial-mesenchymal transition (EMT), and facilitating pathological vascular remodeling. Consequently, targeting the TGF-β1 signaling pathway may represent a potential therapeutic strategy for CMBS.

To achieve the clinical translation of this targeted therapeutic strategy, future research must bridge the gap from basic science to application. We recommend prioritizing the following tasks: Firstly, conduct preclinical studies using animal models to validate the efficacy and safety of TGF-β-targeting drugs. Secondly, at the diagnostic and stratification level, promote research on biomarkers based on TGF-β1/p-Smad3, aiming to establish clinically applicable differential diagnostic criteria and patient stratification models to accurately identify CMBS subgroups suitable for anti-TGF-β1 therapy. Finally, at the therapeutic strategy level, efforts should focus on developing localized drug delivery systems for the airways (e.g., inhaled formulations or intelligent responsive delivery technologies) to maximize therapeutic effects and minimize systemic toxicity, while also exploring combination therapies with anti-inflammatory and anti-fibrotic drugs.

In summary, deepening the understanding of TGF-β1 mechanisms and innovating its targeting strategies hold the promise not only of unraveling the pathological mysteries of CMBS but also of laying a solid foundation for building a precision diagnosis and treatment system for this disease, ultimately improving the prognosis of CMBS patients.
