# Correction: The role of TGF-β1 in chronic multilobar segmental bronchial stenosis and advances in targeted drug research

**DOI:** 10.3389/fphar.2025.1751855

**Published:** 2025-12-03

**Authors:** Mingjun Wu, Qian Yang, Youcheng Xie, Yan Hou, Qingliang Xue

**Affiliations:** 1 Department of Respiratory and Critical Care Medicine, 940th Hospital of Joint Logistic Support Force of Chinese People’s Liberation Army, Lanzhou, Gansu, China; 2 Department of Plateau Medicine, 940th Hospital of Joint Logistic Support Force of Chinese People’s Liberation Army, Lanzhou, Gansu, China; 3 Central Sterile Supply Department, 940th Hospital of Joint Logistic Support Force of Chinese People’s Liberation Army, Lanzhou, Gansu, China

**Keywords:** chronic multilobar segmental bronchial stenosis, transforming growth factor-β1, Smad signaling pathway, targeted therapy, airway remodeling

An incorrect number was provided for the Lanzhou Science and Technology Plan Project. The correct number is 2024-9-167.

The original article has been updated.

